# Efficacy and safety in mice of repeated, lifelong administration of an ANGPTL3 vaccine

**DOI:** 10.1038/s41541-023-00770-3

**Published:** 2023-11-01

**Authors:** Hirotaka Fukami, Jun Morinaga, Hironori Nakagami, Hiroki Hayashi, Yusuke Okadome, Eiji Matsunaga, Tsuyoshi Kadomatsu, Haruki Horiguchi, Michio Sato, Taichi Sugizaki, Keishi Miyata, Daisuke Torigoe, Masashi Mukoyama, Ryuichi Morishita, Yuichi Oike

**Affiliations:** 1 Department of Molecular Genetics, Graduate School of Medical Sciences, Kumamoto University, 1-1-1 Honjo, Chuo-ku, Kumamoto-shi, 860-8556 Japan; 2 Department of Nephrology, Graduate School of Medical Sciences, Kumamoto University, 1-1-1 Honjo, Chuo-ku, Kumamoto-shi, 860-8556 Japan; 3 Center for Metabolic Regulation of Healthy Aging (CMHA), Graduate School of Medical Sciences, Kumamoto University, 1-1-1 Honjo, Chuo-ku, Kumamoto-shi, 860-8556 Japan; 4 Department of Health Development and Medicine, Osaka University Graduate School of Medicine, 2-2 Yamadaoka, Suita-shi, 565-0871 Japan; 5 Department of Aging and Geriatric Medicine, Graduate School of Medical Sciences, Kumamoto University, 1-1-1 Honjo, Chuo-ku, Kumamoto-shi, 860-8556 Japan; 6 Institute of Resource Development and Analysis (IRDA), Kumamoto University, 2-2-1 Honjo, Chuo-ku, Kumamoto-shi, 860-0811 Japan; 7 Department of Clinical Gene Therapy, Osaka University Graduate School of Medicine, 2-2, Yamadaoka, Suita-shi, 565-0871 Japan

**Keywords:** Dyslipidaemias, Drug development

## Abstract

Previously, we reported that an ANGPTL3 vaccine is a hopeful therapeutic option against dyslipidemia. In our current study, we assess durability and booster effects of that vaccine over a period representing a mouse’s lifespan. The vaccine remained effective for over one year, and booster vaccination maintained suppression of circulating triglyceride levels thereafter without major adverse effects on lungs, kidneys, or liver, suggesting vaccine efficacy and safety.

Dyslipidemia is a critical risk factor for atherosclerosis, which increases risk of CVD development and its progression^1,2^. A proportion of patients with dyslipidemia, such as those with familial hypercholesterolemia, are not responsive to drugs commonly used to treat dyslipidemia, such as high-dose statins, ezetimibe, or PCSK9 inhibitors. For these patients, there is an urgent need to develop new medications that counfteract dyslipidemia via different mechanisms^3^.

The secreted protein ANGPTL3 (angiopoietin-like protein 3) blocks lipoprotein lipase activity, thereby worsening lipid profiles including those of circulating low-density lipoprotein cholesterol (LDL-C) and triglyceride (TG). As a result, ANGPTL3 has attracted much attention globally as a new therapeutic target for dyslipidemia, and in 2021, the U.S. Food and Drug Administration approved a monoclonal inhibitory antibody against ANGPTL3 (Evinacumab) as a drug for add-on treatment of adult and pediatric patients ages 12 and above with homozygous familial hypercholesterolemia (HoFH)^4^. Soon after, we established an anti-dyslipidemia peptide vaccine therapy targeting ANGPTL3 [Epitope 3, E3: EPKSRFAMLD (Fig. 1a)] using mouse models of obese dyslipidemia and familial hypercholesterolemia, as a potentially cost-effective therapeutic strategy against dyslipidemia and associated diseases such as fatty liver and atherosclerosis^5^. Our study revealed that increased anti-E3 antibody titers and decreased circulating TG levels in mice in non-fasting conditions were maintained until 30 weeks after the first vaccination in C57BL/6 J wild-type mice, suggesting that vaccine durability was at least half a year^4^. However, some patients with dyslipidemia require long-term treatment with lipid-lowering drugs, often into old age. Thus, there is an urgent need to determine whether immunological memory conferred by our E3 vaccine could be prolonged beyond 6 months and continued until old age and whether booster administration could elevate antibody titers in older animals. In addition, evaluation of vaccine safety was also needed in models of aging mice.

**Fig. 1:**
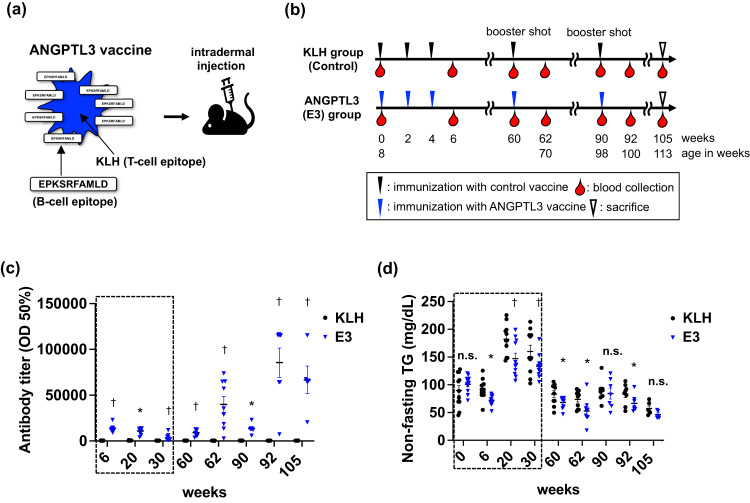
Study protocol and efficacy of the ANGPTL3 vaccine against circulating triglyceride levels in C57BL/6 J mice fed a normal diet. **a** Schematic of vaccine design and vaccination methods. **b** Schematic showing vaccine injection protocol. **c** Circulating antibody titers in mice at 6, 20, 60, 62, 90, 92, and 105 weeks after the first E3 immunization. Values are reported as the serum dilution giving half-maximal binding (optical density: OD50%). *p* < 0.01 for interaction between week and E3 treatment (*n* = 11 per group). Portions of plots within dotted squares represent data referenced from our previous report related to the E3 vaccine^4^. **d** Non-fasting TG levels circulating in mice at 0, 6, 20, 60, 62, 90, 92, and 105 weeks after the first E3 immunization. *p* < 0.01 for interaction between week and E3 treatment (*n* = 11 per group). Portions of plots within dotted squares represent data referenced from our previous report related to the E3 vaccine^4^. **c**, **d** Results are presented as mean ± SEM. n.s. not significant. ∗*p* < 0.05 versus KLH (control) group. ^†^*p* < 0.01 versus KLH (control) group.

Here, we assessed durability of the ANGPTL3 vaccine at one year after the first vaccination and evaluated effects of booster immunization on anti-E3 antibody titers and lipid profiles at 60, 90, and 105 weeks after the first vaccination in C57BL/6 J wild-type mice. We also analyzed safety of the E3 vaccine.

In our previous report, we intradermally vaccinated C57BL/6 J wild-type mice with E3 vaccine 3 times (once every two weeks), starting when mice were 8-weeks-old (Fig. 1b). Mice showed significantly increased anti-E3 antibody titers in circulation and 16.6% lower circulating TG levels by 30 weeks after the first vaccination^5^. In the current study, to assess extended durability of the E3 vaccine, we continued to observe vaccinated mice for a period exceeding 30 weeks. By 60 weeks after the first vaccination, increasing of E3 antibody titers had still been sustained, while circulating TG levels in non-fasting conditions significantly decreased in vaccinated compared to control (KLH) groups (Fig. 1c, d), suggesting that the E3 effect would persist for over a year in mice vaccinated at a young age. However, we noted that the therapeutic efficacy in terms of lowering serum TG levels in non-fasting conditions appeared to weaken between 30 weeks (TG levels decreased 27 mg/dl relative to controls) to 60 weeks (TG levels decreased 15 mg/dl relative to controls) (Fig. 1d), as circulating TG levels decreased in parallel with aging of mice. Accordingly, we performed booster E3 vaccinations at 60 weeks after the first vaccination (Fig. 1b) and found that booster vaccination at this time point markedly increased anti-E3 antibody titers and lowered circulating TG levels in non-fasting conditions relative to controls (Fig. 1c, d), suggesting that the booster was effective. Next, at 90 weeks after the first vaccination, although antibody titers against E3 remained significantly increased in vaccinated versus control groups (Fig. 1c), serum TG levels were comparable in E3 and control groups in non-fasting conditions (Fig. 1d). Moreover, booster E3 vaccination at 90 weeks significantly increased antibody titers and decreased circulating TG levels (Fig. 1c, d). By 105 weeks after the first vaccination, when mice would be near the end of life in a natural setting, the increase in anti-E3 antibody titers remained significant, and circulating TG levels in non-fasting conditions were relatively lower in E3 compared to control groups, although that decrease was not statistically significant (TG levels decreased 11 mg/dl relative to controls, *p* = 0.13) (Fig. 1c). To further analyze lipid profiles, we sacrificed these mice at 105 weeks after 15 h of fasting and found that fasting TG, chylomicron(CM), VLDL-C, LDL-C, sd-LDL-C, HDL-C, and total cholesterol levels in circulation of control mice had decreased with age (to 55.4 mg/dL, 0.43 mg/dl, 7.2 mg/dl, 8.4 mg/dl, 3.9 mg/dl, 44.4 mg/dl, and 61.5 mg/dl, respectively), and that those levels did not differ significantly in vaccinated mice (Supplementary Fig. 1a–g). These data overall suggest that the vaccine is safe without excessive suppression of circulating lipids.

Next, to assess safety of repeated E3 vaccinations on major organs, we examined mouse liver, which is a major source of ANGPTL3 in vivo, as well as kidneys and lungs. Body weight and the liver weight/body weight ratio of the E3 group were comparable to controls throughout the entire protocol, suggesting no significant vaccine toxicity (Fig. 2a, b). Also, circulating levels of alanine aminotransferase (ALT) and aspartate aminotransferase (AST), markers of liver damage, were not significantly elevated in vaccinated versus control mice at 105 weeks after the first vaccination (Fig. 2c, d). Moreover, histological analysis of liver tissue sections revealed no significant signs of inflammation or fibrosis in E3-vaccinated relative to control mice (Fig. 2e–g). Accordingly, transcript levels of markers related to inflammation or fibrosis were comparable in liver tissues from E3 and control groups (Fig. 2h–k). We also observed no major inflammation or fibrosis in sections of kidney and lung tissues in E3-vaccinated compared to KLH control groups, based on histological staining (Supplementary Fig. 2a–f). Renal function was also comparable between these groups (Supplementary Fig. 2g, h). Moreover, we did not observe any overt abnormalities in these organs in vaccinated mice at time of sacrifice, based on inspection of those organs.

**Fig. 2:**
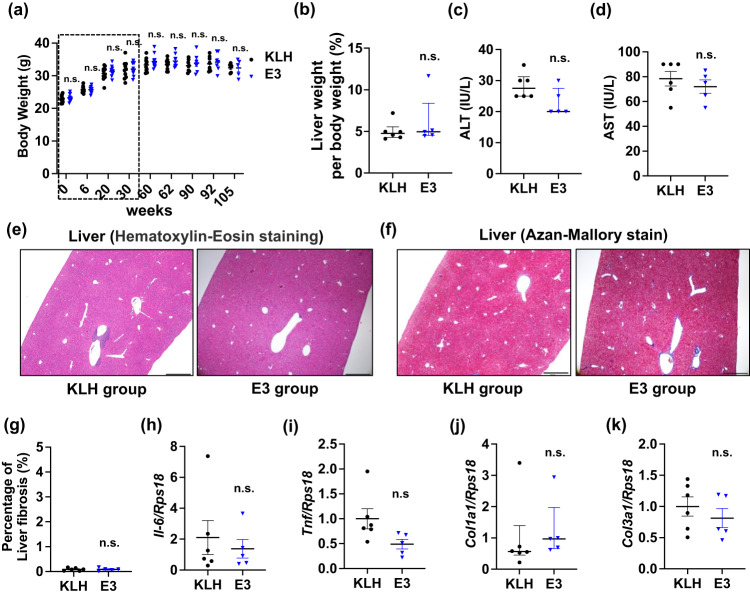
Analysis of parameters relevant to safety after repeated administration of the ANGPTL3 vaccine over the lifespan of mice. **a** Body weight of C57BL/6 J mice before immunization and at 0, 6, 20, 60, 62, 90, 92, and 105 weeks after the first E3 immunization (*n* = 11 per group). All mice were fed a normal diet. Results are presented as mean ± SEM. n.s. not significant. ∗*p* < 0.05 versus KLH (control) group. ^†^*p* < 0.01 versus KLH (control) group. Portions of plots within dotted squares represent data referenced from our previous report related to the E3 vaccine^4^. **b** Liver weight/body weight (%) (KLH: *n* = 6, E3: *n* = 5). **c** Serum alanine aminotransferase (ALT) levels (KLH: *n* = 6, E3: *n* = 5). **d** Serum aspartate aminotransferase (AST) levels (KLH: *n* = 6, E3: *n* = 5). **e** Representative images of Hematoxylin-Eosin-stained liver sections from KLH (left panel) and E3 (right panel) groups. Scale bars, 500 μm. **f** Representative images of Azan-Mallory-stained liver sections from KLH (left panel) and E3 (right panel) groups. Scale bars, 500 μm. **g** The degree of liver fibrosis is reflected by percentages of aniline blue-positive interstitial areas. (H-K) Transcript levels of (**h**) *Il-6*, (**i**) *Tnf-α*, (**j**) *Col1a1* and (**k**) *Col3a1* in liver tissues (KLH: *n* = 5, E3: *n* = 6). KLH group values were set to 1. **a**, **d**, **g**–**i**, **k**) Results are expressed as mean ± SEM. **b**, **c**, **j** Results are expressed as median ± IQR. n.s. not significant; *p* < 0.01 versus KLH (control) group.

Here, we report that intradermal injection of an ANGPTL3 peptide vaccine (E3: EPKSRFAMLD) in C57BL/6 J wild-type mice aged 8, 10, and 12 weeks led to sustained elevation of antibody titers and to a concomitant decrease in triglyceride (TG) levels for up to one year (60 weeks after the initial inoculation). E3 booster administration at weeks 60 and 90 after the primary vaccination markedly increased E3 antibody titers and significantly decreased TG levels in these mice. Notably, treatment with the ANGPTL3 vaccine did not promote adverse suppression of circulating lipids in aging mice or result in major organ damage, suggesting vaccine safety.

In vaccine therapy for inherited chronic diseases such as familial dyslipidemia, it is essential that therapeutic antibodies continue to be produced throughout life. There is concern that the effectiveness of the vaccine may be reduced in older people, as it is thought that the function of memory B cells declines with age, reducing their ability to produce antibodies^6,7^. Hence, doubts have always been raised as to whether vaccine therapy for chronic diseases is realistically feasible. Because to date, few studies in humans and mice have investigated how antibody production after vaccination changes in the long term, into old age, or whether vaccine efficacy can be demonstrated when vaccines are given repeatedly over a lifetime. This study has shown that, at least in mice, repeated doses of our E3 vaccine increase the antibody titers corresponding to the administered antigen over the lifetime of the mice. The results show that vaccine-based treatment strategies are feasible for diseases such as familial dyslipidemia, which require lifelong treatment.

In general, a therapeutic vaccine against lifestyle-related diseases must induce immunity without activating cytotoxic immune responses or promoting adverse autoimmune effects^8^. Notably, although ANGPTL3 is an endogenous, secreted hepatokine synthesized in liver, significant elevation of antibody titers against ANGPTL3 required additional E3 injections, suggesting that autoimmune hepatitis potentially induced by immunoreaction against endogenous ANGPTL3 is of minimal concern. To this end, the B-cell epitope (E3: EPKSRFAMLD) of the ANGPTL3 vaccine was designed to include only 10 amino acids (aa) (corresponding to aa 32–41 of human ANGPTL3 protein)^5^. Based on results reported here, we found that repeated administration of the E3 vaccine throughout life has resulted in safety for major organs.

Mice used here were wild-type male C57BL/6 J fed a normal diet, and the period of analysis extended from young to old age. Both this study and data from the Jackson Laboratory show that serum TG levels in C57BL/6 J mice increase with growth but spontaneously decrease with aging after 26–52 weeks (The Jackson Laboratory’s database)^9^, making it difficult to detect lowering of circulating TG levels and other lipid profiles by the E3 vaccine in older mice that do not exhibit dyslipidemia. We show that E3 vaccination at 8, 10, 12 weeks of age suppressed non-fasting TG levels in circulation for over a year. Furthermore, additional boosting with the vaccine significantly suppressed circulating TG levels in these mice until 100 weeks of age, which is near the end of a mouse’s lifespan. Antibody titers against E3 were high throughout the observation period, indicating that the ANGPTL3 vaccine may induce long-lasting immunological memory. Vaccine administration may be extended and lower vaccine doses may be effective in controlling lipids until old age, possibilities that should be tested in future analyses. Further preclinical studies using genetic dyslipidemia models and obesity-related dyslipidemia models other than C57BL/6 J mice fed a normal diet are needed to test optimal vaccine efficiency. Furthermore, future clinical studies of human subjects treated with the vaccine are also required.

In summary, our anti-dyslipidemic vaccine therapy targeting ANGPTL3 has sustained efficacy for over a year, and additional vaccination restored therapeutic efficacy. Our current study provides insight into the utility and safety of long-term vaccine treatment against lifestyle-related diseases such as dyslipidemia.

## Methods

### Vaccine design and synthesis

We designed a candidate peptide, E3, which corresponds to a region of the LPL inhibitory domain (32–41 aa)^5^. After synthesis, the N-terminal of each peptide was conjugated to KLH, and synthetic peptides were purified by reverse-phase high-performance liquid chromatography (HPLC) (>98% purity) (Peptide Institute, Osaka, Japan). Before immunization, peptide solutions were mixed with an equal volume of complete/incomplete Freund’s adjuvant (FUJIFILM Wako Pure Chemical Corporation, Osaka, Japan).

### Animal studies and immunization

The Ethical Committee approved animal studies based on the Animal Experiments of the Kumamoto University Graduate School of Medical Sciences protocol. Eight-week-old male C57BL/6 J wild-type mice were purchased from Charles River Laboratories (Yokohama, Japan) and bred at 24 °C, in 12-h light-dark cycles, and with free access to normal chow and water. Ten minutes prior to being sacrificed, male mice were anesthetized using a combination of 0.3 mg/kg medetomidine, 4.0 mg/kg midazolam, and 5.0 mg/kg butorphanol, delivered via intraperitoneal injection. Blood was then collected from the tail. Following this, the mice were humanely euthanized through cervical dislocation, and their organs were harvested.

### Evaluation of ANGPTL3 vaccine durability

Eight-week-old male C57BL/6 J wild-type mice (*n* = 11) were intradermally injected with 100 μg KLH-conjugated E3 peptide, followed by identical immunizations 2 and 4 weeks later. Controls were injected with KLH in Freund’s adjuvant following the same protocol. To evaluate vaccine durability, blood samples from mice in non-fasting conditions were collected from the tail vein at 6, 20, 30, 60, 62, 90, and 92 weeks after the first immunization, and antibody titers and non-fasting TG levels were assessed as described below.

### Evaluation of anti-ANGPTL3 antibody titers in immunized mice

Antibody titers were evaluated as described^4^. In brief, ELISA plates (MaxiSorp Nunc; Thermo Fisher Scientific, Waltham, Massachusetts) were coated with 5 mg/mL candidate ANGPTL3 peptides in carbonate buffer overnight at 4 °C. Peptides were conjugated to BSA carrier protein (Peptide Institute). After blocking with PBS containing 5% skim milk, sera were diluted 100- to 312,500-fold in blocking buffer. After overnight incubation at 4 °C and subsequent washing, plates were incubated with horseradish peroxidase (HRP)-conjugated antibodies specific for mouse IgG (GE Healthcare, Chicago, Illinois) for 3 h at room temperature. After washing with PBS, the color was developed using the peroxidase chromogenic substrate 3,3′–5,5′-tetramethyl benzidine (Sigma-Aldrich, St. Louis, Missouri), and the reaction was halted with 0.5 N sulfuric acid. Absorbance at 450 nm was monitored with a microplate reader (iMark, Bio-Rad, Hercules, California). The half-maximal antibody titer was determined according to each sample’s highest value in the dilution range.

### Blood chemistry

Blood TG levels in non-fasting and fasting conditions were measured using a LabAssay kit (FUJIFILM Wako Pure Chemical Corporation) according to the manufacturer’s protocol. Blood ALT, AST, urea nitrogen (UN) and creatinine levels at sacrifice were measured by SRL (Tokyo, Japan), and fasting lipid profile measurements were performed by LipoSEARCH (Immuno-Biological Laboratories) (Gumma, Japan).

### Real-time polymerase chain reaction analysis

Total RNA was extracted using TRIzol reagent (Thermo Fisher Scientific) based on the manufacturer’s protocol. Briefly, DNase-treated RNA was reverse-transcribed using a Prime Script RT reagent Kit (Takara Bio Inc, Shiga, Japan). Quantitative real-time PCR was performed using SYBR Premix Ex TaqII (Takara Bio Inc.). Relative transcript abundance was normalized to that of 18S rRNA levels in mice. Primer sequences are shown in Supplementary Table 1.

### Histological analysis

Paraffin-embedded liver tissue was sliced into 4 μm sections and subjected to Hematoxylin-Eosin or Azan-Mallory staining. The degree of fibrosis was calculated based on the proportion (%) of blue positivity in a defined area in Azan-Mallory-stained sections. All imaging analysis was performed using Photoshop 2022 (version 23.5.5) (Adobe Inc., San Jose, California)

### Statistical analysis

Normality of distribution in continuous variables was evaluated by the Kolmogorov–Smirnov test. If the distribution was normal, data was expressed as means ± SEM. Comparisons between two groups were made using Student’s *t* test. If the distribution was not normal, data was expressed as the median ± interquartile range (IQR), and comparisons between two groups were made using the Mann–Whitney *U* test. A mixed effect model was applied to repeated-measures data using week effects, vaccine effects and interaction of both as explanatory variables. In this model, ID of individual mice was set as a random intercept. A likelihood ratio test was performed to evaluate statistical significance of the interaction. *p* < 0.05 was considered statistically significant. Analyses were performed using GraphPad PRISM version 9.5.1 (GraphPad Software, Inc., La Jolla, California), and STATA MP 17.0 software (StataCorp., College Station, Texas).

### Reporting summary

Further information on research design is available in the Nature Research Reporting Summary linked to this article.

### Supplementary information

Revised supplementary material

REPORTING SUMMARY

## Data Availability

The datasets used and/or analysed during the current study available from the corresponding author on reasonable request.
