# Supplementary material for: Efficacy and safety in mice of repeated, lifelong administration of an ANGPTL3 vaccine
Source: NPJ Vaccines. 2023 Nov 1;8:168. doi: 10.1038/s41541-023-00770-3 (PMC10620388; doi:10.1038/s41541-023-00770-3)
Supplement: Supplementary file 1 — Revised supplementary material [file 41541_2023_770_MOESM1_ESM.docx]

**Supplementary information**

Efficacy and safety in mice of repeated, lifelong administration of an ANGPTL3 vaccine

Hirotaka Fukami, Jun Morinaga, Hironori Nakagami, Hiroki Hayashi, Yusuke Okadome, Eiji Matsunaga, Tsuyoshi Kadomatsu, Haruki Horiguchi, Michio Sato, Taichi Sugizaki, Keishi Miyata, Daisuke Torigoe, Masashi Mukoyama, Ryuichi Morishita, Yuichi Oike

**Supplementary figure**

**
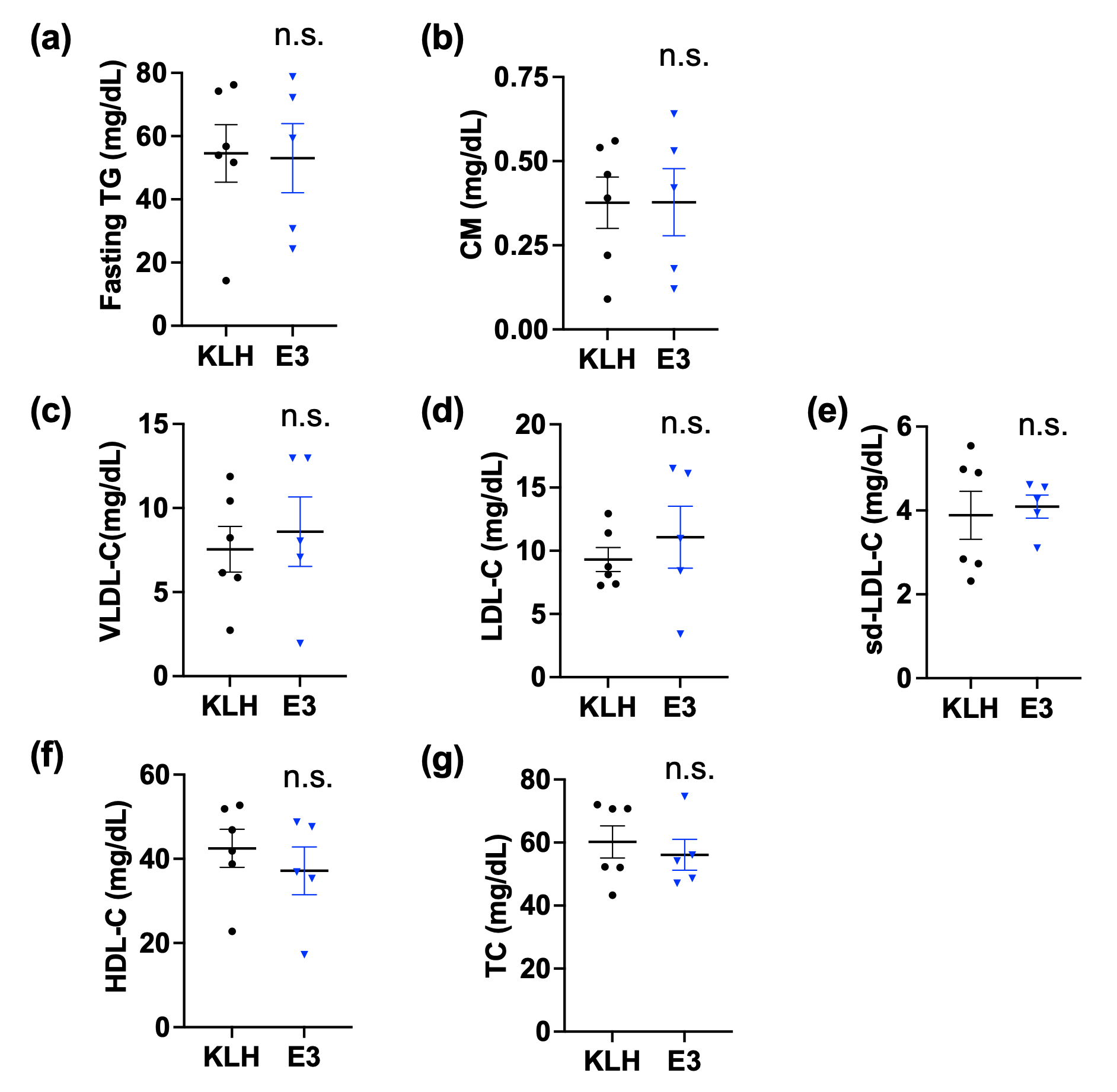
**

Supplementary Figure 1. Evaluation of lipid profiles in C57BL/6J mice at 105 weeks after the first E3 immunization (KLH control: n = 6, E3: n = 5). Levels in fasting animals of (a) triglyceride (TG), (b) chylomicrons (CM), (c) VLDL-C, (d) LDL-C, (e) small dense-LDL-C (sd-LDL-C), (f) HDL-C and (g) TC in circulation. Results are expressed as mean ± SEM. n.s., not significant.


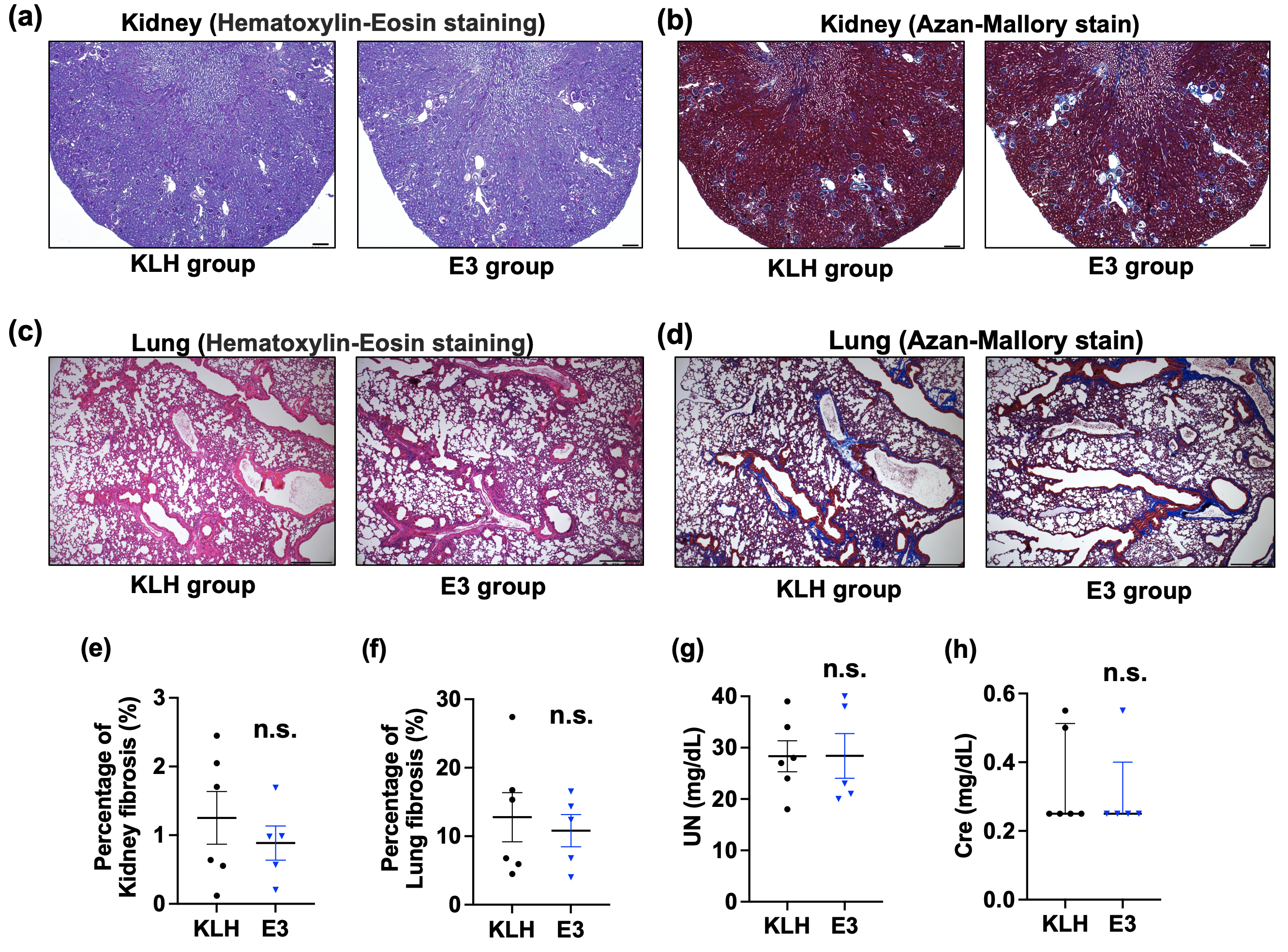


Supplementary Figure 2. Histological analysis of lung and kidney tissue from E3 vaccine-treated versus control mice.

1. Representative images of Hematoxylin-Eosin-stained kidney tissues from

KLH (left panel) and E3 (right panel) groups on week 105 after the initial vaccination. Scale bars, 200 μm. (b) Representative images of Azan-Mallory-stained kidney tissue from groups described in (a). Scale bars, 200 μm. (c) Representative images of Hematoxylin-Eosin-stained lung tissue from KLH (left panel) and E3 (right panel) groups on week 105 after the initial vaccination. Scale bars, 500 μm. (d) Representative images of Azan-Mallory-stained lung tissue in groups described in (c). Scale bars, 500 μm. In all cases, mice were fed a normal diet. (e and f) Percentages of aniline blue-positive interstitial areas indicate the degree of (e) kidney and (f) lung fibrosis (KLH control: n = 6, E3: n = 5). (g) Serum urea nitrogen (UN) levels (KLH control: n = 6, E3: n = 5). (h) Serum creatinine (Cre) levels (KLH control: n = 6, E3: n = 5). (e, f and g) Results are expressed as. mean ± SEM. (h) Results are expressed as median ± IQR. n.s., not significant.

**Supplementary table**

| Gene | Forward primer (5’-3’) | Reverse primer (5’-3’) |
| --- | --- | --- |
| *Rps18* | TTCTGGCCAACGGTCTAGACAAC | CCAGTGGTCTTGGTGTGCTGA |
| *Il-6* | CCACTTCACAAGTCGGAGGCTTA | GCAAGTGCATCATCGTTGTTCATAC |
| *Tnf* | AAGCCTGTAGCCCACGTCGTA | GGCACCACTAGTTGGTTGTCTTTG |
| *Col1a1* | GAGCGGAGAGTACTGGATCGA | CTGACCTGTCTCCATGTTGCA |
| *Col3a1* | CAACCAGTGCAAGTGACCAA | GCACCATTGAGACATTTTGAAG |

Supplementary Table. 1

Sequences of primers of corresponding mouse genes used for quantitative RT-PCR.
